# Exosomes in Ovarian Cancer: Towards Precision Oncology

**DOI:** 10.3390/ph18030371

**Published:** 2025-03-05

**Authors:** Maria Grazia Perrone, Silvana Filieri, Amalia Azzariti, Domenico Armenise, Olga Maria Baldelli, Anselma Liturri, Anna Maria Sardanelli, Savina Ferorelli, Morena Miciaccia, Antonio Scilimati

**Affiliations:** 1 Research Laboratory for Woman and Child Health, Department of Pharmacy—Pharmaceutical Sciences, University of Bari Aldo Moro, Via E. Orabona 4, 70125 Bari, Italy; mariagrazia.perrone@uniba.it (M.G.P.); domenico.armenise1@uniba.it (D.A.); olga.baldelli@uniba.it (O.M.B.); anselma.liturri@uniba.it (A.L.); savina.ferorelli@uniba.it (S.F.); 2 Department of Translational Biomedicine and Neuroscience, University of Bari Aldo Moro, 70124 Bari, Italy; silvana.filieri@uniba.it (S.F.); annamaria.sardanelli@uniba.it (A.M.S.); 3 Laboratory of Experimental Pharmacology, IRCCS Istituto Tumori Giovanni Paolo II, V. O. Flacco, 65, 70124 Bari, Italy; a.azzariti@oncologico.bari.it

**Keywords:** ovarian cancer, exosomes, early diagnosis, theragnostic, treatment

## Abstract

**Background**: Identification of targetable biomarkers to improve early disease detection and overall patient outcomes is becoming an urgent need in clinical oncology. Ovarian cancer (OC) has one of the highest mortality rates among gynecological cancers. It is asymptomatic and almost always diagnosed at an advanced stage (III or IV), leading to a 5-year survival rate of approximately 35%. **Methods**: Current therapeutic approaches for OC are very limited and mainly consist of cytoreductive surgery and cisplatin plus taxane-based chemotherapy. No gender and tumor specific biomarkers are known. Exosomes, lipid bilayer vesicles of endocytic origin secreted by most cell types, represent sources of information for their involvement in the onset and progression of many diseases. Hence, research on exosome contents as tools and targets in precise oncology therapy provides knowledge essential to improving diagnosis and prognosis of the disease. **Results**: This review attempts to give an overview of how exosomes are implicated in ovarian carcinoma pathogenesis to trigger further cancer exosome-based investigations aimed at developing ovarian cancer fine-tuning diagnostic methodologies. **Conclusions**: It is essential to investigate exosome-based cancer drugs to advance understanding, improve treatment plans, create personalized strategies, ensure safety, and speed up clinical translation to increase patients’ overall survival and quality of life. Papers published in PubMed and Web of Science databases in the last five years (2020–2024) were used as a bibliographic source.

## 1. Ovarian Cancer

Ovarian cancer (OC) is the seventh most frequently diagnosed cancer and the fifth leading cause of cancer-related mortality in women worldwide. According to ovarian cancer estimates by the American Cancer Society, approximately 19,680 new cases and 12,740 deaths due to this disease were expected in 2024 [1]. The absence of early symptoms, the lack of a reliable screening test [2], and the presence of non-specific signs that can be mistaken for benign conditions contribute to late-stage diagnosis (FIGO stage III or IV) in over 70% of ovarian cancer cases. For these reasons, OC is often referred to as the “silent killer.”

Ovarian cancer is rare in pediatric patients, accounting for only 5% of all OC cases globally, and is uncommon in young women. However, the risk significantly increases in women over 50 [3,4]. Although initial treatment, consisting of radical surgery followed by platinum- and taxane-based chemotherapy, often has high response rates, most patients eventually experience a relapse, with a median disease-free survival of approximately 18 months [5]. The overall five-year survival rate remains around 35%. Once platinum-resistant recurrences occur, treatment options become limited, with additional chemotherapy agents achieving response rates of only 15–20% and a median progression-free survival of approximately 4 months.

The most prevalent form of OC originates from epithelial cells (EOC) and can be classified into four distinct histological subtypes: serous, clear cell, endometrioid, and mucinous [6]. Additionally, EOC is further divided into low-grade and high-grade EOC. High-grade serous ovarian cancer (HGSOC) accounts for 70–80% of all EOCs and represents the most aggressive form, characterized by significant heterogeneity and genomic instability. This malignancy typically arises in the thin layer of tissue covering the outer surface of the ovaries [7,8,9].

Currently, the only FDA-approved biomarkers for ovarian cancer detection are serum carbohydrate antigen 125 (CA125) and human epididymis protein 4 (HE4). However, these markers lack specificity for both gender and disease type, with limited predictive value due to their low sensitivity, leading to false positives in approximately 50% of cases. Elevated CA125 levels have also been observed in various physiological and pathological conditions, such as menstruation, pregnancy, endometriosis, and peritoneal inflammatory diseases. In contrast, HE4 levels appear to be influenced by smoking [10,11], with increases of 20–30% observed in female smokers [12,13,14]. At present, the most effective diagnostic approach involves combining CA125 and HE4 levels to assess the risk of ovarian cancer in patients suspected of having benign conditions, such as cystadenofibroma [15]. The development of a set of highly specific and sensitive biomarkers could significantly improve the prognosis of OC by enabling earlier detection and more effective treatment strategies.

## 2. Exosomes

**Figure 1:**
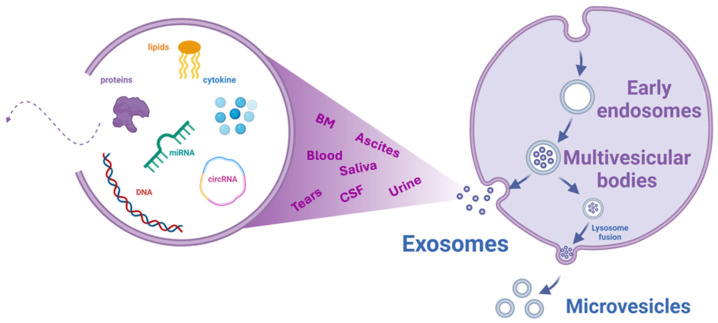
Schematic representation of exosome biogenesis, secretion, and molecular content release. Exosomes protrude from the surface of the membrane and, degraded by lysosomes or secreted as multivesicular bodies, release their cargo in various body fluids, e.g., saliva, blood, breast milk (BM), tears, urine, cerebrospinal fluid (CSF), and ascites. Created with Biorender.com.

Among the emerging biomarkers in oncology, extracellular vesicles (EVs) have gained considerable attention [16]. These vesicles, which include ectosomes, microvesicles, microparticles, exosomes, oncosomes, and apoptotic bodies, differ in their biogenesis, size, function, and release mechanisms. Due to the lack of distinct markers for each subpopulation, the term “EV” is generally used, as recommended by the International Society of Extracellular Vesicles (ISEV) [17]. Among these, exosomes—small extracellular vesicles ranging from 30 to 150 nm—are of particular scientific and clinical interest. These vesicles, secreted by living cells through an endocytic pathway, play a crucial role in intercellular communication by transporting nucleic acids and bioactive molecules between cells [18]. Their formation involves the fusion of surface membrane invaginations (multivesicular bodies) with vesicles originating from the Golgi apparatus, highlighting their importance in cell signaling and disease progression (Figure 1) [19].

Exosomes are found in various bodily fluids, including blood, urine, saliva, breast milk (BM), tears, cerebrospinal, and ascitic fluid, where they function as transporters of bioactive molecules such as lipids, proteins, RNAs, double-stranded DNA, non-coding RNAs, and microRNAs (miRNAs). These extracellular vesicles play a crucial role in mediating communication between tumor cells and their surrounding microenvironment, influencing the progression and behavior of malignant cells [20].

The release of exosomes is regulated by several factors, including changes in microenvironment pH as well as the activity of oncogenes and tumor suppressors, which can modulate exosome secretion in cancer [21,22].

Recent studies have highlighted the role of exosomes derived from ovarian cancer cells in disease progression, particularly in facilitating peritoneal dissemination and contributing to the establishment of a pre-metastatic niche [23].

Exosomes have emerged as a promising tool for real-time monitoring of tumor biology in vivo, owing to their high bioavailability, stability, biocompatibility, and ability to carry molecular cargo [24]. The transfer of information from exosomes to target cells occurs through three primary mechanisms: (1) receptor–ligand interactions, (2) direct fusion with the plasma membrane, and (3) endocytosis via phagocytosis. Researchers investigating exosomes focus on identifying the most precise methods for their isolation and characterization, as well as understanding their potential as biomarkers for diagnosis, prognosis, and predicting responses to anti-tumor therapies. Additionally, exosomes are being explored as therapeutic agents for cancer treatment and as nanocarriers for drug delivery.

These extracellular vesicles (EVs) hold significant potential for the development of personalized cancer treatments. However, despite their well-established role in disease progression, efforts to target exosome biogenesis, release, and uptake as therapeutic (neo)adjuvants remain limited.

**Figure 2:**
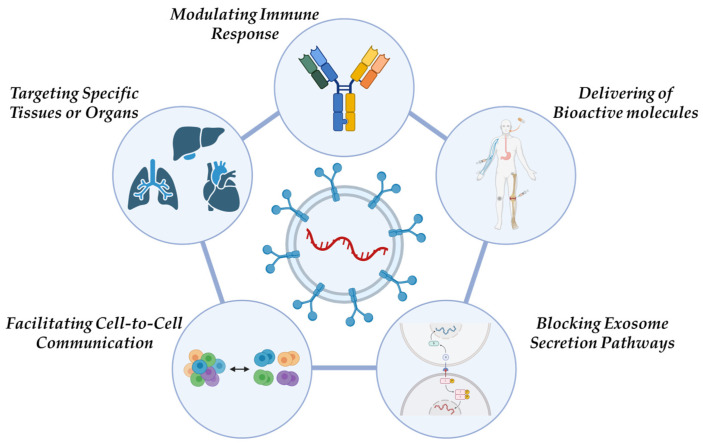
Strategies for leveraging exosomes in cancer therapy. Created with Biorender.com.

Currently, several strategies are being explored to leverage exosomes in cancer therapy, including the following (Figure 2) [25]:

- ***Blocking Exosome Secretion Pathways:*** Cancer cells exploit exosome secretion to affect the tumor microenvironment, enhance tumor growth, drive invasion, and develop resistance to treatment. A potential therapeutic approach involves either blocking the mechanisms responsible for exosome dissemination within malignant cells or removing EVs from the blood circulatory system.
- ***Delivering Bioactive molecules:*** Exosomes, due to their lipid membrane composition, efficiently facilitate cellular uptake of bioactive molecules. This makes them ideal carriers for anticancer drugs, miRNAs, and siRNAs. Additionally, they can transport tumor antigens, nanobodies, apoptosis-inducing proteins, proteasomes, mutated or deficient anti-apoptotic proteins, tissue-specific peptides, transferrins, and lactoferrins to tumor cells.
- ***Targeting Specific Tissues or Organs:*** Due to their intrinsic cell tropism, exosomes can selectively target specific tissues or organs, offering a promising approach for precision medicine.
- ***Modulating Immune Responses:*** Exosomes play a role in immune system regulation, which has potential applications in the development of cancer vaccines aimed at slowing or preventing tumor progression.
- ***Facilitating Cell-to-Cell Communication***: Exosomal miRNAs originating from tumor cells contribute to intercellular communication, influencing various cellular processes related to cancer progression.

### 2.1. Exosome Isolation and Purification Strategies

Exosomes can be isolated from both cell cultures and biological fluids using various techniques and methodologies (Table 1). Several approaches are available for their identification and quantification, including nucleic acid sequencing (DNA sequencing), quantitative real-time PCR (qRT-PCR), enzyme-linked immunosorbent assay (ELISA), and magnetic-activated cell sorting. Western blotting and flow cytometry are commonly used to identify exosomes utilizing immune-based isolation with antibody-conjugated beads targeting exosome surface antigens. Exosome isolation can be achieved using different techniques (Table 1).

#### 2.1.1. Sucrose Density Gradient Ultracentrifugation

Ultracentrifugation, combined with sucrose density gradients or sucrose cushions to separate low-density exosomes, is considered the gold standard for exosome isolation. However, this method is highly labor-intensive and time-consuming, requiring up to two days per preparation when using sucrose gradients. Moreover, it demands substantial biological sample volumes while yielding relatively low quantities of exosomes [26,27]. The process involves multiple high-speed ultracentrifugation steps to eliminate cellular debris and concentrate exosomes. When combined with sequential centrifugation in a sucrose gradient, this technique enables the purification of high-quality exosome preparations.

#### 2.1.2. Polymer-Based Precipitation

This technique uses hydrophilic polymers like polyethylene glycol to attract water molecules surrounding exosomes, creating a hydrophobic environment that reduces solubility and promotes precipitation. The sample is then subjected to low-speed centrifugation to collect EVs. Compared to ultracentrifugation, this technique offers higher yields starting from smaller sample volumes or multiple biological specimens while minimizing excessive centrifugal forces that could potentially damage exosomes, offering a simpler and faster alternative for exosome isolation. Several commercial kits have been developed for EV extraction based on this method. However, one drawback is the potential formation of protein aggregates, which could interfere with sample purification and quality [27].

#### 2.1.3. Ultrafiltration

This method enables the isolation of exosomes based on their size without considering their electrical charge. However, the difficulty with this technique is the removal of residual particles that stick to the nanomembrane, leading to lower efficiency [28].

#### 2.1.4. Size-Exclusion Liquid Chromatography (SEC)

Like ultrafiltration, SEC allows exosome isolation by size. Since body fluids and cell culture media contain various nanoparticles that have the same size range as exosomes, SEC is particularly useful. Exosome recovery is improved by combining ultrafiltration and SEC [29]. However, the main limitations of these techniques include their time-consuming nature, the risk of lipoprotein contamination, and the potential formation of protein aggregates. Additionally, these methods are not suitable for isolating exosomes from membrane particles, large protein aggregates, or apoptotic vesicles, as they fall within the same size range as exosomes [30].

#### 2.1.5. Immunomagnetic Beads and Nanoparticle Tracking Analysis (NTA)

Exosomes can be isolated using immunomagnetic microspheres, where the spheres bind to the exosomes. After separation, the exosomes are recovered from a chip and analyzed using transmission electron microscopy (TEM). The results achieved by on-chip isolation are then validated and compared with those obtained from exosomes isolated by ultracentrifugation using NTA, which helps determine the size distribution and concentration of small particles in liquid [31].

#### 2.1.6. Immunoaffinity System

A commonly used method for exosome extraction is a modified version of magnetic cell sorting that uses anti-epithelial cell adhesion molecule (EpCAM) antibodies. The process involves immunoaffinity capture using anti-EpCAM-coated magnetic microbeads to pre-enrich exosomes from the serum of patients with benign conditions and early-stage ovarian cancer. Although this immunoaffinity method is promising for diagnostics and therapy, it is most effective when many exosomes express the target protein [32,33].

#### 2.1.7. Microfluidic Systems

Recent advancements in exosome research have merged microfluidic systems developed to miniaturize devices, lower costs, and minimize required biological sample volumes while maintaining high separation efficiency [34]. They include the following:

- Microfluidic immunoaffinity separation, which mimics traditional bead-based approaches by embedding antibodies within microfluidic channels to selectively capture exosomes. While highly specific, this method requires traditional RNA extraction, unlike bead-based systems that allow direct miRNA retrieval. Kabe et al. introduced a microfluidic system incorporating immunomagnetic beads for enhanced exosome isolation from plasma, demonstrating high diagnostic efficacy in ovarian cancer through multiplexed tumor marker detection [34];
- Filtration using microfluidic chips with nanomembranes or nanowires offers a straightforward approach for exosome isolation, relying on size exclusion. This technique includes the use of porous polymer monoliths in a microfluidic system with DC electrophoresis to prevent clogging. Moreover, the development of the Exosome Total Isolation Chip (ExoTIC), a modular platform designed to isolate exosomes from urine, plasma, and cell culture media, achieves yields up to 1000-fold higher than ultracentrifugation within three hours. While it offers significant efficiency, the complexity of nanowire-based structures may present challenges in clinical implementation [35,36];
- Deterministic Lateral Displacement (DLD) classifies particles based on size using micro-structured columns that guide smaller particles through predefined trajectories while redirecting larger ones. Nano-DLD arrays have successfully separated particles ranging from 20 to 110 nm with high precision. However, traditional DLD systems require high hydrodynamic resistance, necessitating pressures exceeding 200 kPa. Wu et al. optimized this method by incorporating electro-osmotic flow, significantly reducing the required pressure while maintaining continuous and precise separation [37];
- Acoustic-based exosome isolation is a label-free approach that utilizes ultrasound standing waves to achieve high selectivity and biocompatibility. Acoustic forces act on particles based on their volume, allowing for precise size-based separation. Recent developments have integrated acoustics with microfluidics into a two-module system: the first step removes large blood components, while the second isolates exosomes, achieving 82.4% recovery and 98.4% purity. This method provides a streamlined, high-purity exosome isolation process directly from whole blood with minimal processing [38];
- Electrokinetic approaches, such as di-electrophoresis (DEP) and electrophoresis (EP), leverage alternating current (AC) voltage to separate exosomes. DEP exploits differences in particle polarization in a non-uniform electric field, while EP directs charged particles based on their electrophoretic mobility. An alternating current electrokinetic microarray chip has been developed to isolate exosomes from plasma in 30 min by attracting them to high-field regions while larger particles migrate to low-field areas [39];
- Viscoelastic microfluidic separation is a label-free technique that exploits differential elastic lift forces among particles of varying sizes. Due to their small size, exosomes experience minimal viscoelastic effects. Wang et al. developed a microfluidic system with two inlets and three outlets, where polyoxyethylene polymers are used to enhance the viscoelasticity of exosomes. In this system, larger particles exit through the central outlet, while exosomes are collected at the side outlets. This approach achieves high separation purity (>90%) and recovery (80%) without the need for complex procedures [40].

The increasing interest in exosomal miRNAs as disease biomarkers has driven the rapid development of commercial isolation kits, offering simple and efficient protocols with minimal sample requirements. Some kits yield higher miRNA concentrations from serum-derived exosomes than ultracentrifugation. However, most commercial kits isolate exosomes via precipitation, leading to the co-isolation of other EVs and protein complexes. To optimize circulating exosome miRNA analysis, evaluating the performance of these commercial kits in clinical applications remains essential [41].

## 3. Exosomes Isolated from Ovarian Cancer: Content and Role in Tumor Malignancy

In ovarian cancer, exosomes play a significant role in tumor growth by regulating angiogenesis, cell proliferation, and migration. These vesicles carry bioactive molecules that are transferred to endothelial cells and other components of the tumor microenvironment, thereby promoting tumor progression, metastasis, and drug resistance.

Exosomes derived from ovarian cancer (OC-exosomes) have the potential to serve as both biomarkers and therapeutic targets for the disease. Several methods have been developed to identify OC-specific exosomes. Advances in exosome membrane component analysis and electromagnetic technology have contributed to the creation of highly sensitive devices for detecting OC-associated exosomes [34]. The A8 peptide aptamer, which targets membrane heat shock protein 70 (HSP70), has been utilized to extract exosomes from urine and blood samples of OC patients [42,43]. A commonly employed approach for visualizing OC-derived exosomes involves labeling them with the lipophilic fluorescent dye PKH67 for flow cytometry analysis [44]. Additionally, electrochemical sensors and microfluidic chip-based platforms detect exosomes in body fluids [45].

A graphene oxide/polydopamine (GO/PDA) nano-interface has been developed for highly accurate exosome detection in OC patients, enabling quantification from minimal plasma sample volumes [46]. TEM remains the gold standard for imaging extracellular vesicles, including exosomes, and specialized software has recently been introduced to analyze their morphological characteristics in TEM images. Additionally, advances in high-throughput sequencing have sped up the analysis of genetic and epigenetic markers in exosomes from different biological fluids, improving the early detection and characterization of OC [47]. Exosome membrane proteins like CD24 and EpCAM can be detected in ascitic fluid from ovarian cancer patients using nano-plasmonic sensors. Nanoparticle tracking analysis revealed a higher concentration of exosomes in the ascitic fluid compared to the blood plasma, which is abundant in ovarian cancer patients and contains high levels of cytokines, chemokines, and growth factors that facilitate tumor progression. Exosomes found in ascites express tumor-associated antigens such as Mart1 and HER2/neu, which can activate cytotoxic T lymphocytes (CTLs), suggesting their potential application in immunotherapy [48]. Beyond carrying growth-promoting signals, these exosomes modulate immune responses by influencing NF-κB and STAT3 signaling pathways in lymphocytes. Moreover, they impair the ability of natural killer (NK) cells to eliminate tumor cells, contributing to immune evasion.

## 4. Ovarian Cancer-Specific Exosome Cargo

### 4.1. Lipidomic Content of OC-Exosomes

Lipidomic analysis of exosomes derived from ovarian cancer cell lines SKOV-3 and HOSEPiC cell cultures identified 1227 lipid species and 30 lipid classes. A significant difference in lipid content was observed between exosomes from these two cell lines. SKOV-3-derived exosomes exhibited higher levels of monosialodihexosylganglioside (GM3), zymosterol ester, lysophosphatidylinositol, lysophosphatidylserine, and cholesterol ester. Moreover, exosome lipids are being explored as potential biomarkers for tumorigenesis. For example, CerG3 was exclusively detected in HOSEPiC-derived exosomes, whereas phosphatidylserine was specifically found in SKOV3-derived exosomes. This lipid specificity presents a promising method for identifying novel diagnostic biomarkers in liquid biopsy since lipid composition of OC-exosomes closely resembles that of the plasma membrane of the cell from which they originate. The most common lipids found in exosomes include phosphoglycerolipids, sphingolipids, and sterols. Compared to their parent cells, exosomes are particularly enriched in phosphatidylserine, disaturated phosphatidylethanolamine, disaturated phosphatidylcholine, sphingomyelin, ganglioside GM3, and cholesterol. However, the lipid profile of exosomes can vary among different subpopulations. For instance, the lipid composition of CD61-positive exosomes differs significantly from that of other subtypes [49,50].

### 4.2. Proteomic Content of Exosomes

Exosomes from the blood plasma and ascitic fluid of OC patients have been shown to carry proteins involved in carcinogenesis, including ATF2, MTA1, ROCK1/2, and CD147, which contribute to tumour angiogenesis, and GNA12, EPHA2, and COIA1, which promote migration and metastasis. Furthermore, exosomes carry heat shock proteins (Hsp90 and Hsc70), MHCI, and MHCII, along with enzymes such as phosphate isomerase, peroxiredoxins, aldehyde reductase, and fatty acid synthase. Recent research has demonstrated that exosome levels are significantly higher in OC patients compared to healthy donors, along with an increase in the expression of LRP1, a protein from the low-density lipoprotein receptor family. LRP1 regulates signaling pathway activation and controls MMP2 and MMP9 expression, both of which influence OC cell migration in vitro and in vivo.

Proteomic analysis of exosomes from SKOV3 and HOSEPiC cell cultures identified 659 universal proteins among a total of 1,433 exosome proteins. COX2, one of the most abundant exosome proteins, showed increased expression under hypoxic conditions, and its overexpression is linked to tumour spheroid formation and metastasis. A comparative proteomic study of blood plasma exosomes from 43 OC patients and 46 healthy donors revealed 294 universal proteins, with 69 unique proteins found in the OC patient group.

Up to 40% of the unique proteins in ascitic fluid are found in exosomes, including proteins such as CD24, EpCAM, CD171, MMP-2, MMP-9, uPA, MT1-MMP, ADAM10, ADAM7, ADAM17, CD151, and TSPAN8. These proteins mediate epithelial–mesenchymal transition (EMT), premetastatic niche formation, and peritoneal dissemination, and their overexpression has been correlated with advanced disease stage. The formation of premetastatic niches is a crucial mechanism in OC progression, with tumour-secreted exosomes playing an essential role in this process. Exosomes from OC patients’ ascites also contain higher concentrations of STAT3 and FAS, proteins that contribute to carcinogenesis and tumour dissemination [50].

**Figure 3:**
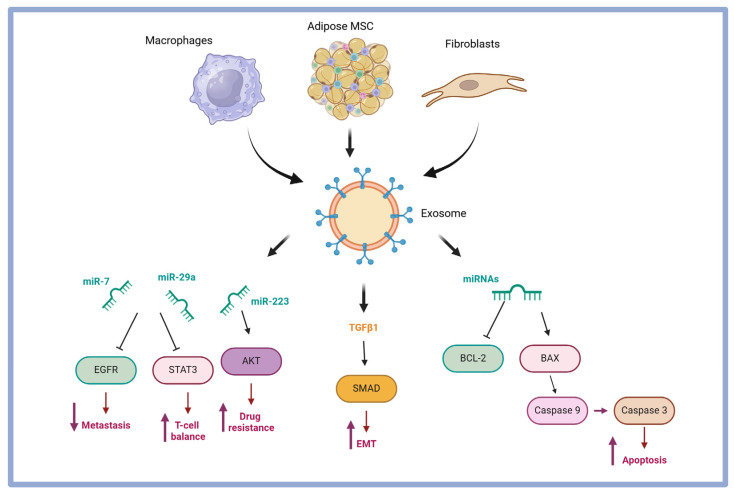
Exosomal microRNAs released by adipose-derived mesenchymal stem cells (MSCs), macrophages, and fibroblasts in ovarian cancer cells. miRNAs released by adipose MSCs induce apoptosis by decreasing BCL-2 and increasing BAX expression that induce Caspase-3 activation increasing apoptosis. miR-7, miR-29-a, and miR-223 reduce metastasis, induce T-cell balance, and induce drug resistance, respectively. TGFβ1 induces EMT via the SMAD pathway. Created with Biorender.com.

Beyond promoting tumor growth, exosomes also help ovarian cancer evade immune surveillance. They can convert normal fibroblasts into cancer-associated fibroblasts (CAFs), which release exosomes rich in TGFβ1, which enhances tumor cell migration and invasion while activating the SMAD signaling pathway, further promoting cell migration and invasion (Figure 3).

### 4.3. RNAs Transported by Exosomes

#### 4.3.1. Long Non-Coding RNAs (lncRNAs)

LncRNAs, typically longer than 200 nucleotides, are highly variable and can bind competitively to proteins or mRNAs. They play a crucial role in cancer development, angiogenesis, metastasis, and drug resistance. A study comparing exosomes from OC patients, benign ovarian tumors, and healthy women found alterations in the expression of 425 lncRNAs, including notable changes in long intergenic non-protein coding RNAs (e.g., LINC00470, LINC01811, LINC02343, and LINC02428, FER1L6 antisense RNA 2, CXXC4 Antisense RNA 1) that promote tumor growth, migration, and invasion.

In OC-exosomes derived from cisplatin-resistant A2780 cells, lncRNA MEG3, which inhibits carcinogenesis and reduces drug resistance, was downregulated. Additionally, lncRNAs ENST00000444164 and ENST0000043768, found in OC-exosomes from SKOV-3 cells, were shown to play a key role in endothelial cell migration and phosphorylation of NF-κB in HUVEC cultures, although further studies are needed to fully understand the NF-κB pathway regulation.

The exosome lncRNA MALAT1 was found in OC patient sera, and its elevated levels correlated with aggressive disease and metastasis [51].

A higher level of lncRNA αHIF was observed in exosomes from OC patients compared to healthy controls, and it is linked to a more aggressive disease and poorer survival, making it a potential biomarker for OC progression.

In summary, various lncRNAs found in exosomes are implicated in cancer progression, resistance to treatment, and metastasis. These lncRNAs and their interactions with proteins involved in crucial biological pathways present potential biomarkers for diagnosis and therapeutic targets in OC.

#### 4.3.2. microRNAs (miRNAs)

MiRNAs transported by exosomes are critical mediators of OC progression. Under hypoxic conditions, certain miRNAs, such as miR-222-3p, promote the formation of blood and lymphatic vessels (angiogenesis and lymphangiogenesis) [52,53]. Others, including miR-940, miR-21-3p, miR-125b-5p, and miR-181d-5p, drive macrophages toward a tumor-supportive M2 phenotype by regulating the suppressor of cytokine signaling (SOCS)4/5/signal transducer and activator of transcription 3 (STAT3) pathways and enhancing cancer cell proliferation and migration. Conversely, miR-146b-5p, also carried by tumor-derived exosomes, regulates endothelial cell migration by modulating TRAF6, a protein involved in inflammation, immune response, and apoptosis [54].

Several studies indicate that EVs affect prognostic mechanisms, such as the downregulation of Programmed Cell Death 4 (PDCD4), a gene involved in apoptosis, which is directly regulated by oncogenic miR-21. Reduced PDCD4 expression has been linked to OC progression and poor prognosis, alongside miR-21 overexpression [55]. Furthermore, increased levels of serum exosome miR-100, miR-200b, and miR-320 are associated with shorter survival times and advanced disease stages (Stage III and IV), indicating their potential role as diagnostic and prognostic markers [56].

Another relevant finding is the upregulation of miR-30a-5p in urine from OC patients compared to healthy women. This molecule appears to be more abundant in early-stage OC (Stage I–II) compared to more advanced stages (Stage III–IV), suggesting a potential oncogenic function. Similarly, analysis of serum miR-1290 expression suggests its utility in early detection, particularly for high-grade serous ovarian cancer (HGSOC) [57]. miR-34b plays a crucial role in suppressing epithelial–mesenchymal transition (EMT) in OC by targeting Notch2, downregulating E-cadherin, and upregulating N-cadherin and Snail, thereby mitigating OC progression (Table 2) [58].

#### 4.3.3. circularRNAs (circRNAs)

CircRNAs appear to be stable and enriched in exosomes, making them detectable in blood circulation and urine from OC patients (Table 3) [59].

The median expression level of Circular RNA ITCH (circ-ITCH) is significantly lower than in adjacent non-cancerous tissues and negatively correlated with tumor size and FIGO staging in OC [60]. Another circRNA, circABCB10, has been linked to poor overall survival (OS), although it is not considered an independent predictive factor for OC. Studies indicate that circ-ABCB10 is upregulated and negatively regulates miR-1271, miR-1252, and miR-203 in OC [61].

CircRNA1656 expression is correlated with FIGO staging in OC patients, making it a potential tumor marker [62]. Similarly, high circHIPK3 expression has been associated with lymph node metastasis, advanced FIGO stage, and poorer disease-free survival (DFS) and OS rates [63].

The low expression levels of circLARP4 in OC suggest that they are associated with advanced FIGO stages and lymphonode metastasis, indicating its potential role as a prognostic biomarker [64].

**Table 3:** Emerging circRNAs in ovarian cancer pathogenesis.

CircRNA	Regulation in OC	Prognosis
**circ-ITCH**	down	poor OS [60]
**circ-ABCB10**	up	poor OS [61]
**circ-1656**	up	poor OS [62]
**circHIPK3**	up	poor OS/poor disease-free survival (DFS) [63]
**circLARP4**	down	poor OS/poor disease-free survival (DFS) [64]

Despite the identification of various lipid, protein, and RNA components in OC-exosomes, their precise biological functions remain unclear. Developing clinically useful exosome biomarkers using small amounts of bodily fluids could improve OC diagnosis; additionally, understanding the mechanisms behind exosome release within the tumor microenvironment as well as from OC-derived exosomes could be crucial for designing strategies for preventing OC metastasis [65].

## 5. Exosomes as Drug Delivery Systems for Treating Ovarian Cancer

Exosomes have emerged as promising nanocarriers for drug delivery due to their natural organotropism and exceptional stability in the bloodstream. Advances in exosome-based therapies have enabled the development of molecularly targeted treatments. Their biocompatibility, structural integrity, nanoscale size, cargo-carrying capacity, and modifiable surface make them ideal for therapeutic applications. Researchers have explored various strategies for efficiently integrating bioactive agents, including drugs, nucleic acids, proteins, peptides, and nanomaterials, into these vesicles.

Two main strategies are used for drug delivery: passive and active loading. Passive loading relies on diffusion-assisted methods such as co-incubation with exosomes or exosome-secreting cells, transfection (often enhanced by electroporation), sonication, freeze–thaw cycles, dialysis, extrusion, surfactant treatment, and in situ synthesis. Such physical techniques facilitate cargo loading by inducing micropores in the exosome membrane, enhancing permeability. However, these techniques may compromise exosome membrane integrity, potentially affecting targeting efficiency. Passive diffusion is the simplest method, relying on the incubation of exosomes or their producing cells with therapeutic agents. This process requires isolating exosomes from biological fluids, tissues, or cultured cells, followed by incubation under controlled conditions. This method has been successfully applied to mesenchymal stromal cells exposed to paclitaxel-released drug-loaded exosomes showing tumor-inhibitory effects [66]. Similarly, metastatic ovarian and breast cancer cells treated with paclitaxel and doxorubicin produced exosomes carrying these drugs, effectively suppressing tumor progression. However, passive loading has limitations, including low efficiency and difficulty in precisely controlling cargo concentration. To enhance loading efficiency, transfection is widely used to introduce nucleic acids, proteins, and peptides into exosomes. This involves inserting plasmids into cells to induce the expression of target molecules, which are then included in exosomes. A more advanced method, Targeted and Modular EV Loading (TAMEL), improves RNA encapsulation by anchoring RNA-binding domains onto exosome-enriched proteins, allowing selective cargo incorporation. While TAMEL shows potential, further research is needed to address clinical challenges [67].

**Figure 4:**
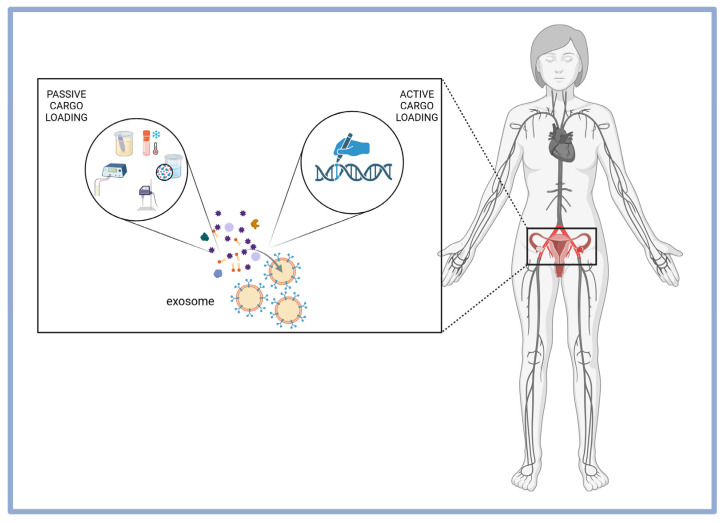
Two main strategies for OC-specific exosome drug delivery: passive and active. Passive loading relies on diffusion-based methods like co-incubation and other physical treatments to enhance exosome permeability, e.g., electroporation, sonication, freeze–thaw cycles, dialysis, extrusion, surfactant treatment, and in situ synthesis. Active loading leverages cellular mechanisms to incorporate proteins or nucleic acids during exosome biogenesis, often through genetic modification of parental cells. Created with Biorender.com.

Active loading, in contrast, leverages cellular mechanisms to incorporate therapeutic proteins or nucleic acids during exosome biogenesis. This can be achieved by genetically modifying parental cells to overexpress specific bioactive molecules, which are subsequently packaged into exosomes (Figure 4). This method involves fusing cargo proteins with exosome membrane proteins, as demonstrated in technologies developed by System Biosciences and Evox Therapeutics [68]. However, challenges remain, particularly when the fusion of cargo proteins with exosome membranes alters their biological function. To address these limitations, ILIAS Biologics has introduced an innovative platform known as Exosomes for Protein Loading via Optically Reversible Protein–Protein Interaction (EXPLOR). This system enhances protein loading efficiency while mitigating structural and functional disruptions associated with previous methods [68].

Unlike synthetic drug carriers, exosomes offer unique advantages due to their biological origin and natural role in intercellular communication. However, their use in drug delivery presents challenges for large-scale clinical implementation. Key considerations include selecting appropriate donor cells, optimizing therapeutic cargo (such as small interfering RNAs, microRNAs, or pharmaceutical compounds), improving loading techniques, designing targeted exosome modifications, and determining the most effective administration route. A potential solution to streamline these challenges and enhance clinical applications is the development of exosome mimetics—synthetic liposomes engineered to incorporate essential components of natural exosomes while overcoming scalability and reproducibility issues. The rapid expansion of exosome research over the last decade has highlighted their immense potential as diagnostic and therapeutic biomarkers [69], as well as targets for various cancers, with particular interest in their role in ovarian cancer therapy, drug resistance, and their capacity to enhance patient survival [70]. Given the aggressive nature of ovarian cancer and the frequent development of resistance to conventional chemotherapy, there is an urgent need for novel therapeutic strategies that can improve patient outcomes. In this context, precision medicine approaches [71,72,73], leveraging advanced molecular profiling and personalized treatment regimens, may offer new avenues to enhance the efficacy of exosome-based drug delivery, providing more targeted and effective therapies tailored to the specific genetic and molecular characteristics of each patient.

## 6. Conclusions

Recent studies on ovarian cancer, particularly on biomarkers and chemotherapy, published in PubMed over the past five years have highlighted the significant potential of exosomes in both diagnostic and therapeutic applications. Investigating the contents of exosomes derived from cancer cells could be crucial in assessing disease status and treatment effectiveness while also offering insights into tumor progression and drug resistance mechanisms. Exosomes, reflecting the characteristics of their parent cells, are ideal biomarkers for cancer diagnosis and targeted therapy due to their stability in circulation and ease of collection from body fluids.

The characteristics of exosomes from cancer cells are also linked to tumor progression and invasion by overcoming the immune response and enhancing multi-drug resistance, often through the elimination of chemotherapeutic drugs via membrane efflux pumps like P-gp. Advancements are needed in the large-scale production of exosomes from suitable donor cells, efficient loading of therapeutic agents, and the optimization of targeted delivery to specific tissues. Advancements in developing strategies to produce many exosomes from suitable donor cells, efficiently load therapeutic agents into exosomes, and optimize the targeted delivery of exosomes to specific tissues will facilitate their use as natural carriers in clinical therapy. These innovations will be essential for using exosomes as natural carriers in clinical settings. The ultimate goal to reduce recurrence rates, increase the progression-free interval, and improve overall survival rates for five years post-diagnosis remains a significant challenge in ovarian cancer today. With continued research and technological innovations, exosomes could transform the landscape of cancer knowledge and treatment, offering a promising avenue for precision medicine.

## Figures and Tables

**Table 1:** Summary and comparison of different exosome isolation techniques.

Methods		Advantages	Disadvantages
**Sucrose density gradient ultracentrifugation**		Efficient isolation of exosomes of different densities, most commonly used, easy approach	Labor and time-consuming, special equipment required (ultracentrifuge), low efficiency
**Polymeric precipitation**		Small sample, simple one-step method	Formation of protein aggregates
**Ultrafiltration**		High exosome purity, faster, easy to handle compared to ultracentrifugation	Deformation/breaking up of vesicles (splat factor), filtration rate may be affected by pore size and sample concentration
**Size-exclusion liquid chromatography (SEC)**		High exosome purity, efficacy to remove debris and contaminants	Labor and time consuming, sample contamination with lipoproteins, formation of protein aggregates, membrane damage or disruption during isolation may impact exosome function and properties
**Immunomagnetic beads**		Efficient isolation of exosomes, potential for downstream analysis	Low levels of the target protein
**Nanoparticle tracking analysis**		Ideal for small particles	Accuracy depends on exosome content
**Immunoaffinity system**		Fast, easy to use, no specialized equipment	Exosome separation with targeted protein only
**Microfluidic systems**	Filtration	Small volumes of biological samples, reduced isolation time	Requires staff competent in the microfluidic platform
	Deterministic Lateral Displacement (DLD)		
	Acoustic-wave-based device		
	Electrical-field-based device		
	Viscoelastic-flow based		

**Table 2:** Summary of miRNAs released by OC-exosomes.

miRNA	Function	Mechanism	Clinical Implications
**miR-222-3p**	Angiogenesis, lymphangiogenesis	Promotes blood and lymphatic vessel formation under hypoxic conditions	Enhances OC progression [52]
**miR-940, miR-21-3p, miR-125b-5p, miR-181d-5p**	Macrophage polarization	Drive macrophages toward a tumor-supportive M2 phenotype, enhancing cancer cell proliferation and migration	Create a tumor-promoting microenvironment [53]
**miR-146b-5p**	Endothelial cell migration	Modulates TRAF6, regulating inflammation, immune response, and apoptosis	Influences tumor progression by affecting cell migration [53]
**miR-21**	Apoptosis regulation	Suppresses PDCD4, reducing programmed cell death	Associated with poor prognosis and tumor progression [55]
**miR-100, miR-200b, miR-320**	Prognostic markers	Increased levels in patients with advanced OC (Stage III–IV)	Correlated with reduced survival and advanced disease stage [56]
**miR-30a-5p**	Early diagnosis	More abundant in early-stage OC (Stage I–II) compared to advanced stages (Stage III–IV)	Potential biomarker for early OC detection [56]
**miR-1290**	Early diagnosis	Highly expressed in serum, especially in high-grade serous ovarian cancer (HGSOC)	Possible tool for early OC detection [57]
**miR-34b**	Early diagnosis	Inhibitory effect on cell proliferation and epithelial–mesenchymal transition (EMT)	Potential biomarker for early OC detection [58]
